# A Mixed Micelle Formulation for Oral Delivery of Vitamin K

**DOI:** 10.1007/s11095-016-1954-9

**Published:** 2016-05-31

**Authors:** Feilong Sun, Tessa C. C. Jaspers, Peter M. van Hasselt, Wim E. Hennink, Cornelus F. van Nostrum

**Affiliations:** 1 Department of Pharmaceutics, Utrecht Institute for Pharmaceutical Sciences, Utrecht University, 3584 CG Utrecht, The Netherlands; 2 Department of Pediatrics, Wilhelmina Children’s Hospital, University Medical Center Utrecht, Lundlaan 6, 3584 EA Utrecht, The Netherlands

**Keywords:** Konakion® MM, Mixed micelles, PEGylation, pH stability, Vitamin K

## Abstract

**Purpose:**

To develop a stable micellar formulation of vitamin K for oral delivery, because the commercial and clinically used formulation of vitamin K (Konakion® MM) destabilizes at gastric pH resulting in low bioavailability of this vitamin in neonates with cholestasis.

**Methods:**

Mixed micelles composed of EPC, DSPE-PEG 2000 and glycocholic acid, with and without vitamin K, were prepared by a film hydration method. The influence of pH on the stability of the micelles was analyzed by dynamic light scattering (DLS). The critical micelle concentration (CMC) was determined by fluorescence spectroscopy using pyrene and the morphology was evaluated by transmission electron microscopy . Caco-2 cells were used to study the cytocompatibilty.

**Results:**

Mixed micelles with mean diameters from 7.1 to 11.0 nm and a narrow size distribution (PDI < 0.2) were obtained after 3 membrane extrusion cycles. Konakion® MM formed aggregated particles at gastric pH, which was avoided through steric stabilization by introducing PEG. TEM showed that mixed micelles had a spherical size (diameter of around 10 nm) with a narrow size distribution in agreement with the DLS results. The loading capacities for vitamin K of mixed micelles with varying molar fractions of DSPE-PEG and EPC (from 0/100 to 50/50 (mol/mol)) were 10.8–5.0 w%, respectively. The mixed micelles showed good cytocompatibility at concentrations of glycocholic acid between 0.12 and 1.20 mM.

**Conclusions:**

Mixed micelles with superior stability to Konakion® MM at low pH were obtained by introducing DSPE-PEG 2000. These are therefore attractive oral formulations for vitamin K.

**Electronic supplementary material:**

The online version of this article (doi:10.1007/s11095-016-1954-9) contains supplementary material, which is available to authorized users.

## INTRODUCTION

Vitamin K is an essential cofactor for γ-glutamyl carboxylase, an enzyme that catalyzes the carboxylation of glutamic acid residues in a number of proteins that are involved in the blood coagulation (1). In healthy adults, bile that is secreted into the intestine facilitates the solubilization, transport and uptake of vitamin K present in food (2). Therefore, dietary deficiency of vitamin K is rare in adults, except for patients suffering from intestinal disorders and malabsorption (3). On the other hand, vitamin K deficiency does frequently occur in neonates due to limited transport of vitamin K through the placental barrier and low concentration in breast milk (main exogenous source of vitamin K in neonates) (4). This in turn is associated with the risk of life-threatening vitamin K deficiency bleeding (VKDB), which is due to the low activity of vitamin K-dependent coagulation factors (II, VII, IX, X) (5). Konakion® mixed micelles (MM) Paediatric is a formulation composed of vitamin K, egg phosphatidylcholine (EPC or lecithin) and glycocholic acid (6). It is indicated for both the prophylaxis and treatment of VKDB in neonates as well as infants. However, orally administered Konakion® MM fails to prevent VKDB in cholestatic infants due to impaired intestinal absorption of vitamin K (7), which is due to the pathophysiological conditions in the upper gastrointestinal tract of those infants. Glycocholic acid has a carboxylic acid group with a pKa of 3.8 (8), which ensures a good colloidal stability of the mixed micelles above pH > ~4. However, Konakion® MM is unstable and forms large aggregates at low pH of the stomach because of the protonation of the carboxylate group of glycocholic acid, eventually causing coalescence of the formulation (9). After stomach passage, large aggregates of lipids and coalesced vitamin K enter the duodenum, which under normal physiological conditions can subsequently be solubilized by endogenous bile salts (10,11), pancreatic lipases and elevated pH above the pKa of glycocholic acid (12,13). On the other hand, once large aggregates are formed in the stomach of neonates suffering from cholestasis, coalesced vitamin K cannot be solubilized due to the very low level of bile salts in these patients (13,14).

Mixed micelles composed of phospholipids and bile salts were first described by Hoffman and Borgstrom (15) as associated amphiphilic biliary and dietary components. The hydrophobic tails of EPC molecules are positioned in the core of the mixed micelles to minimize their contact with water molecules while their polar head groups are present at the interface with water (16). It has been further shown that phospholipids self-assemble into bilayered structures in aqueous solution, whereas mixed micelles are formed in the presence of bile salts (16). These structures can solubilize poorly water-soluble drugs in the inner micellar core (17). Furthermore, mixed micelles are thermodynamically stable with sizes usually ranging from 5 to 60 nm (18). Particles with such small diameters are necessary to transport vitamin K to the surface of enterocytes and to retain mixed micelles at the base of microvilli (19).

The aim of this study was to develop a mixed micellar formulation of vitamin K that is stable at low pH and therefore suitable for oral administration. Therefore, part of EPC of the Konakion® MM formulation was substituted by a PEGylated lipid (DSPE-PEG 2000). The rationale behind this idea is that PEGylated phospholipids are known to act as a steric barrier stabilizing nanostructures such as liposomes (20,21), micelles (22), disks (23) and dendritic nanocarriers (24), and will thereby enhance the stability of drug loaded formulations at neutral as well as acidic pH. For example, Torchilin *et al.* have shown that DSPE-PEG 2000/D-α-tocopheryl PEG 1000 succinate (TPGS) micelles are stable at low pH (25). PEGylated lipids have so far not been a component of endogenous lecithin/bile salt mixed micelles to enhance their stability at low pH. Yet, those bile salt-lipids based mixed micelles expose unique possibilities to enhance the permeability of hydrophobic drugs in the intestine (26). Furthermore, EPC, glycocholic acid and DSPE-PEG 2000 are all components approved by U.S. FDA. Therefore, to mimic the endogenous mixed micelles, a mixed micellar formulation based on above components for vitamin K is preferred instead of for instance polymeric micelles, which can also remain stable at acidic condition.

The size and pH stability of mixed micelles composed of varying ratios of DSPE-PEG/EPC were analyzed by dynamic light scattering (DLS). Extrusion effects on the size of mixed micelles during filtration were investigated using DLS and TEM. The loading capacity of vitamin K for mixed micelles composed of DSPE-PEG /EPC 50/50 (mol/mol) was optimized. Furthermore, cell viability studies were conducted with Caco-2 cells to evaluate the cytocompatibility of the mixed micelles.

## MATERIALS AND METHODS

### Materials

Lecithin (egg phosphatidylcholine, EPC) and 1,2-distearoyl-sn-glycero-3-phosphoethanolamine-poly(ethylene glycol) 2000 (DSPE-PEG 2000) were kindly provided by Lipoid GmbH (Ludwigshafen, Germany). Potassium phosphate monobasic (KH_2_PO_4_) and sodium phosphate dibasic dihydrate (Na_2_HPO_4_ · 2H_2_O) were purchased from Sigma-Aldrich (Zwijndrecht, The Netherlands) and used to prepare 0.067 M phosphate buffer (0.35 g KH_2_PO_4_ and 1.45 g Na_2_HPO_4_ · 2H_2_O in 100 ml RO-water, pH 7.3). Sodium chloride were purchased from Merck KGaA (Darmstadt, Germany). Sodium phosphate monobasic monohydrate (NaH_2_PO_4_ · H_2_O) and Pepsin from porcine stomach mucosa was provided by Sigma-Aldrich (Zwijndrecht, The Netherlands). Vitamin K, glycocholic acid hydrate, fetal bovine serum (FBS) and all other chemicals and reagents were obtained from Sigma-Aldrich (Zwijndrecht, The Netherlands). Chloroform was provided from Biosolve (Valkenswaard, the Netherlands). Sodium hydroxide (NaOH), 70% perchloric acid, hexa-ammoniummolybdate ((NH_4_)_6_ M_O7_O_24_ · H_2_O) and ethanol were supplied by Merck KGaA (Darmstadt, Germany). *N*-Methyl dibenzopyrazine methylsulfate (PMS) and sodium 3′-[1-(phenylaminocarbonyl)-3,4-tetrazolium]-bis(4-methoxy-6-nitro)benzene sulfonic acid hydrate) (XTT) were provided from Sigma. Dulbecco’s Modified Eagle’s Medium (DMEM) and RPMI 1640 medium were purchased from GibCo BRL Life Technologies (Carlsbad, CA, USA). Syringe filters were obtained from Phenomenex (0.2 μm, Torrance, CA). Ultrapure water was produced by a Synergy UV water delivery system from Millipore (Billerica, MA, USA). Konakion® MM was a product manufactured by Roche (Basel, Switzerland).

## METHODS

### Preparation of Empty Micelles

Various amounts of EPC and DSPE-PEG 2000 were dissolved in 4 ml chloroform in a round-bottom flask (Table I). The solvent was evaporated under vacuum at 60°C for 20 min to form a film. Next, glycocholic acid hydrate (110 mg) was dissolved in 8 ml 0.067 M phosphate buffer at 60°C and the obtained solution was subsequently added to hydrate the above mentioned film of EPC and DSPE-PEG 2000. A transparent dispersion was obtained after magnetically stirring for at least 4 h at room temperature. The dispersion was subsequently extruded 3 times through a syringe filter.

**Table I:** Mixed Micelles Composed of DSPE-PEG, EPC and Glycocholic Acid

Component	Formulations			
	A	B	C	D
DSPE-PEG 2000 (mmol)	–	0.02	0.06	0.10
EPC (mmol)	0.20	0.18	0.14	0.10
DSPE-PEG/EPC (mol/mol)	0/100	10/90	30/70	50/50

The amounts of glycocholic acid and total lipids in the formulations were 0.24 and 0.20 mmol, respectively

### Preparation of Vitamin K Loaded Micelles

Vitamin K (1 g) was dissolved in 10 ml chloroform. Next, 0.2 ml of this vitamin K solution was added to 3.8 ml chloroform in which the different amounts of lipids (EPC and DSPE-PEG 2000) were dissolved (Table I). The solvent was evaporated under vacuum at 60°C for 20 min to form a film. Next, glycocholic acid hydrate (110 mg, 0.24 mmol) was dissolved in 8 ml 0.067 M phosphate buffer at 60°C and the obtained solution was subsequently added to hydrate the above mentioned film of EPC, DSPE-PEG 2000 and vitamin K. A transparent dispersion was obtained after magnetically stirring for at least 4 h at room temperature. The dispersion was subsequently extruded 3 times through a syringe filter. The dispersions were covered with aluminized foil to protect Vitamin K against light-triggered degradation during the whole procedure (27).

To investigate the vitamin K loading capacity of the DSPE-PEG/EPC 50/50 mixed micelles, DSPE-PEG (288 mg, 0.10 mmol) and EPC (80 mg, 0.10 mmol) were dissolved in 4 ml chloroform in a round-bottom flask. Next, 0.4 or 0.8 ml of vitamin K stock solution (100 mg/ml in chloroform) was added. The following steps were the same as described above until a transparent dispersion was obtained. Then the obtained micellar dispersions were further diluted ten times with 0.067 M phosphate buffer before filtration to avoid occulation of the filter. Subsequently, the dispersions were extruded 6 times through a filter.

### Characterization of Mixed Micelles

Average size and size distribution of the different mixed micelles dispersed in 0.067 M phosphate buffer were measured by Dynamic Light Scattering (DLS; Zetasizer 4000, Malvern Instruments, Malvern, UK) at 25°C and at an angle of 90°. The morphology of the mixed micelles was studied by Transmission Electron Microscopy (TEM, Tecnai 10, Philips, and 100 kV). The samples for TEM visualization were prepared according to the following procedure. A sample of the mixed micelles dispersion (10 μl) was pipetted onto parafilm and a carbon-coated copper grid subsequently was placed on the sample for 4 min. Next, the excess liquid was removed by filter paper and subsequently the grid was negatively stained by being placed on a 10 μl droplet of 2% uranyl acetate in demineralized water for 1 min. Next, the excess liquid was removed using a filter paper and the grid was dried for 5 min at room temperature before the measurement. The zeta-potential (ζ potential) of the mixed micelles was determined by Zetasizer (Malvern Instruments Ltd.). Prior to the measurements, phosphate buffer was exchanged by 10 mM Hepes buffer pH 7.4 using PD-10 column chromatography. The instrument was calibrated using a standard (DTS1235, −42.0 ± 4.2 mV, Malvern Instruments, UK).

### Determination of the Critical Micelle Concentration (CMC)

The CMC of the mixed micelles was determined using pyrene as a fluorescent probe (28). In detail, empty micelles composed of EPC only and DSPE-PEG/EPC (50/50, mol/mol, formulations A and D, Table I) were prepared as described in the section “Preparation of Empty Micelles”. Next the micellar dispersions were diluted with 0.067 M phosphate buffer to different concentrations from 5.9 × 10^−6^ to 29.5 mM glycocholic acid (and 5.2 × 10^−6^ to 25.8 mM total lipids). Next, 18 μl of pyrene in acetone (1.8 × 10^−4^ M) was added to 4.5 ml of each micellar dispersion. The micellar dispersions with pyrene were incubated for 20 h at room temperature in the dark to allow evaporation of acetone. Fluorescence excitation spectra of pyrene were recorded using a Horiba Fluorolog fluorometer (at a 90° angle). The excitation spectra (300 to 360 nm with emission wavelength of 390 nm) were recorded at 37°C. The excitation and emission band slits were 4 and 2 nm, respectively. The excitation intensity ratio of I_338_/I_333_ was plotted against the concentration of glycocholic acid to determine the CMC, which was obtained as the point of intersection of two tangents drawn to the curve at high and low concentrations, respectively (29).

### Stability of the Micelles

Two milliliter of vitamin K loaded micellar dispersions composed of various amounts of DSPE-PEG/EPC (see Table I, prepared as described in section “Preparation of Vitamin K Loaded Micelles”) were diluted 2.5 times by addition of 3 ml 0.067 M phosphate buffer. For pH stability study, the pH of the micellar dispersions was stepwise lowered from 7.2 to 1.6 by addition of 1 M HCl. After each step, the dispersions were incubated for 5 min at 25°C and the size of the micelles was measured by DLS at the same temperature. Subsequently, the pH of dispersions was raised stepwise from 1.6 to 7.0 by adding 1 M NaOH and after each step the dispersions were incubated for 5 min at 25°C before measuring the size of the micelles. For determination of vitamin K recovery, the pH of the mixed micelles was lowered to 1.6 with 1 M HCl and the dispersions were subsequently incubated for 1 h at 37°C. Subsequently, the dispersions were centrifuged at 1000×*g* for 1 min and 0.1 ml of the different supernatants was diluted with 1 ml ethanol to dissolve the mixed micelles and the vitamin K content was measured by HPLC as described below.

To mimic gastric stability at *in vivo* conditions, fasted state simulated gastric fluid (FaSSGF) was used, without bile salts (30). 1.50 ml mixed micelles composed of EPC only (Konakion®MM) and DSPE-PEG/EPC (50/50, mol/mol) with different concentrations of vitamin K (from 0.625 to 5.0 mg/ml) was added to 0.75 ml FaSSGF (20.0 μM lecithin, 34.2 mM NaCl and 0.1 mg/ml Pepsin, pH 1.5) and 0.24 ml 1 M HCL (to adjust pH to 1.5). The mixture was incubated at 37°C for 1 h with slow rotating.

### Determination of Vitamin K by HPLC Analysis

The concentration of vitamin K in the dispersions of the different mixed micelles was determined by RP-HPLC after dilution with ethanol. A SunFire C_18_ column was used and absorption at 254 nm was used for detection. The mobile phase was ethanol/water (95/5, *v*/*v*). The column temperature was 30°C. The injection volume was 20 μl. A calibration curve was obtained using vitamin K dissolved in ethanol with concentrations ranging from 19.8 to 181.8 μg/ml.

### Determination of Lipid Recovery

The lipid recovery was determined using a method based on the colorimetric determination of PO_4_^3−^ according to Rouser (31). The phospholipid content of the mixed micelles was determined after destruction of the phospholipids with perchloric acid (HCLO_4_) to yield inorganic phosphate. Standard solutions were prepared as follows: 20/40/60/80/100/120/160 μl of 0.5 mM phosphate solution (1.95 g NaH_2_PO_4_^.^H_2_O dissolved in 250 ml of RO-water and subsequently diluted 100 times with RO-water) was introduced in triplicate in test-tubes. Test samples were prepared by dilution of the micellar dispersions 2.5 times with RO-water and 1.8 ml of the diluted dispersion was subsequently desalted using PD-10 column chromatography (Pharmacia). The collected volume was 2.0 ml. Phosphate buffer (1.8 ml, 0.067 M) was also desalted using PD-10 column chromatography as control. Then, samples of the micellar dispersions and the control sample were 10 times diluted with RO-water. Next, samples of 190 μl of the diluted micellar dispersions and the control were pipetted in triplicate into the test tubes. The tubes were covered by porcelain marbles and subsequently heated using a block heater (Techne Dri-block heater, Model DB 3H, PJ Brennan, Dublin) for 30 min (temperature was set at 180°C). Next, the test tubes were cooled down to room temperature and 0.3 ml of 70% perchloric acid was introduced in the tubes. The tubes were again covered by porcelain marbles and heated by the block heater (temperature at 180°C) for at least 45 min until the solutions were clear. Next, 1 ml RO-water, 0.5 ml 1.2% hexa-ammoniummolybdate solution (1.25 g hexa-ammoniummolybdate in 100 ml of RO-water) and 0.5 ml of 5% (*w*/*v*) ascorbic acid (1.00 g in 20 ml of RO-water) were added to the test tubes. Subsequently, the tubes were covered by the porcelain marbles and placed in a water bath (100°C) for 5 min. After cooling down to room temperature, the absorbance of the solutions at 797 nm was determined using a UV–vis spectrophotometer (UV mini-1240, Shimadzu, Japan) and the amount of phosphate in the samples was determined using the calibration curve as described above.

### Cytocompatibility of Vitamin K Loaded Mixed Micelles

The viability of Caco-2 cells incubated with vitamin K loaded mixed micelles was evaluated by the XTT assay (32). The cells were seeded in 96-well plates at a density of 1 × 10^4^ cells per well and incubated at 37°C with 5% CO_2_ for 24 h to permit cells attachment. Because glychocholic acid is the main component of the formulation which can cause cytotoxicity (33), the concentration of the mixed micelles is reported in terms of glycocholic acid content. Konakion® MM and vitamin K loaded mixed micelles (2.5 mg/ml) composed of EPC only and DSPE-PEG/EPC (10/90, 30/70 and 50/50, mol/mol, respectively) were diluted with Dulbecco’s Modified Eagle’s medium (blank DMEM). The final concentrations of glycocholic acid of the mixed micelles were from 0.12 to 1.20 mM after addition of 20 μl samples to 100 μl DMEM culture medium. The control was empty micelles composed of DSPE-PEG/EPC (50/50, mol/mol) (final concentration of glycocholic acid was 1.2 mM). To investigate the viability of caco-2 cells at higher concentrations of mixed micelles, formulations composed of DSPE-PEG/EPC (50/50, mol/mol) and EPC only were used. They were diluted with Dulbecco’s Modified Eagle’s medium (blank DMEM) and their final concentrations of glycocholic acid were from 0.12 to 12 mM. 100 μl of above mixed micelles was added to each well. After 24 h of culturing at 37°C, the medium was removed and 50 μl XTT solution (1 mg/ml in plain RPMI 1640, containing 25 μM PMS) was added per well and the plates were subsequently incubated for 1.5 h at 37°C, after which the absorbance at 490 nm with a reference wavelength of 655 nm was measured by ELISA microplate reader (Biorad Novapath).

## RESULTS AND DISCUSSION

### Preparation of Mixed Micelles with and Without Vitamin K Loading

**Fig. 1:**
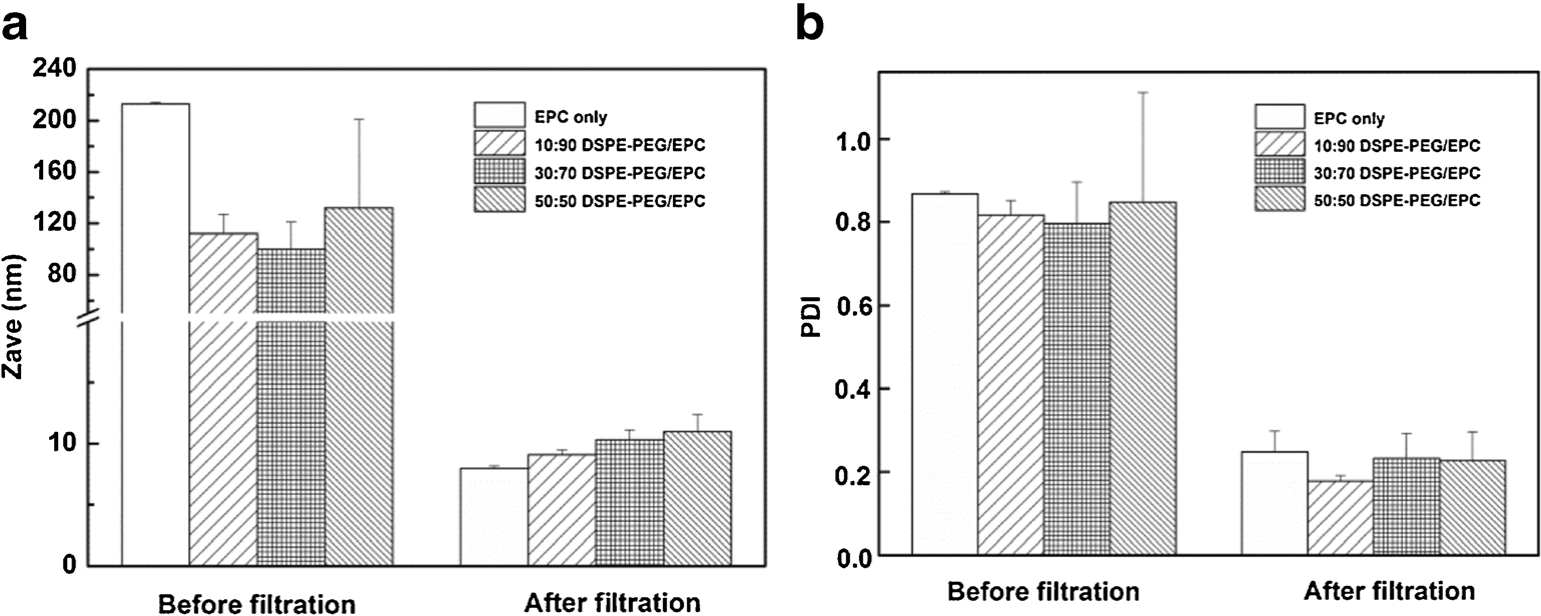
Z-average diameter (**a**) and polydispersity index (PDI) (**b**) of vitamin K loaded mixed micelles composed of different molar ratio of DSPE-PEG/EPC before and after filtrations.

Vitamin K loaded mixed micelles composed of EPC, with and without DSPE-PEG, and a fixed molar ratio of 0.22 vitamin K *versus* total lipids, were prepared by hydration of a lipid film with a glycocholic acid solution, resulting in turbid dispersions probably consisting of large unilamellar liposomes coexisting with flat bilayers as has been reported earlier (34,35). The turbid dispersions became gradually translucent upon magnetic stirring, and finally transparent micellar dispersions were obtained after stirring for 4 h. The average size of the dispersed particles after stirring for 4 h and before filtration was between 85 and 215 nm (Fig. 1a) with high polydispersity (Fig. 1b). Such high polydispersity suggests the coexistence of micelles and larger vesicles.

**Fig. 2:**
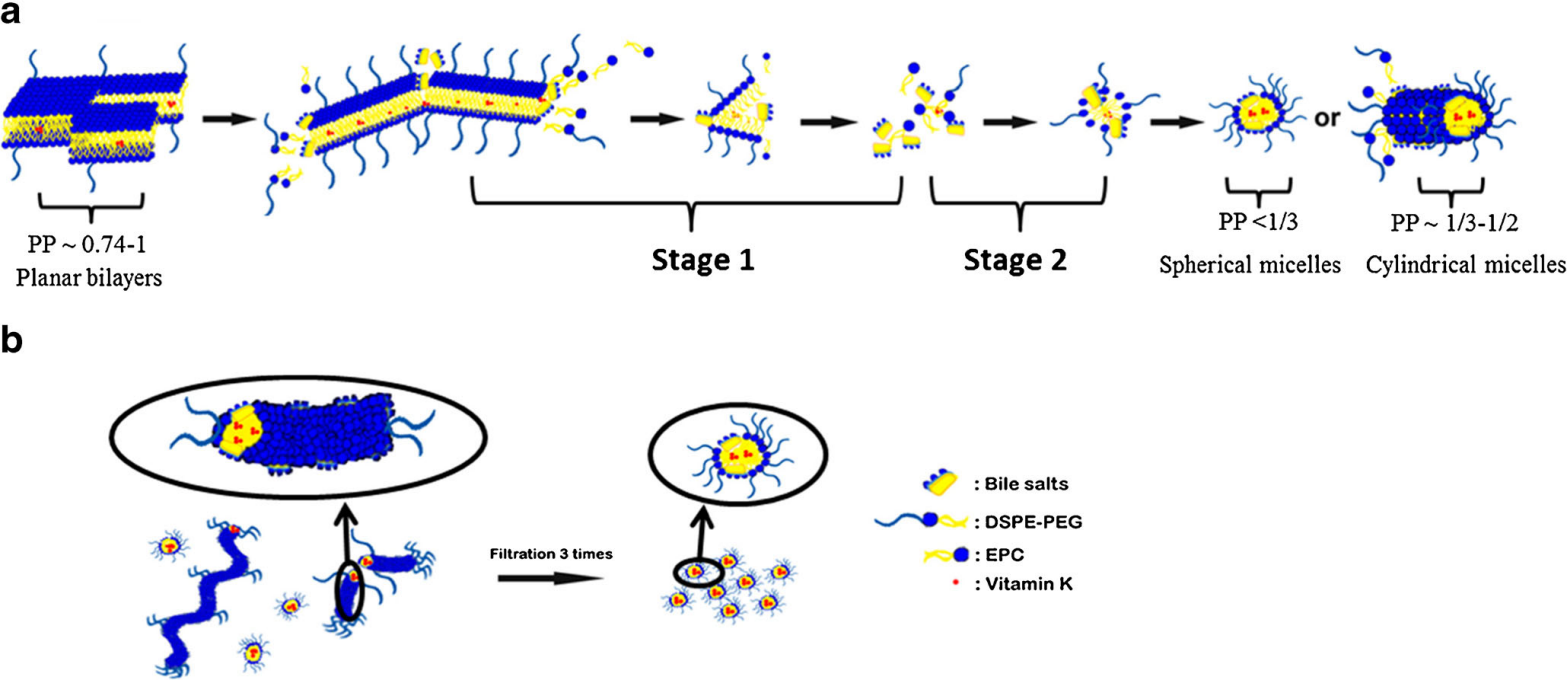
A schematic mechanism of the formation of micelles from planar phospholipid bilayers during hydration of a film in a bile salt solution under stirring before filtration (**a**), and the size reduction after repeated filtrations through a 0.2 μm filter (**b**).

There are at least two factors involved in the change from turbid to transparent dispersions. First, the molecular volume (V) of the phospholipid EPC is approximately equal to the polar surface cross-area (A_o_) and the length (L) of the hydrophobic chain, meaning that the packing parameter PP = V/ (A_o_ × L) (36), is close to 1 and as a consequence this phospholipid tends to form planar bilayers (37). The insertion of glycocholic acid in the phospholipid vesicles, due to its flat structure, can increase A_o_ which results in a reduction of the PP. As a consequence, part of the vesicles is transformed into highly curved mixed micelles (38) as shown in Fig. 2a. This glycocholic acid induced vesicles-to-micelles transition process occurs in three steps (39). Glycocholic acid initially partitions between water and the outermost planar bilayer and subsequently penetrates and peels off the planar lipid bilayers one after the other yielding smaller bilayer flakes (stage 1). Subsequently, after being peeled off, phospholipid molecules and glycocholic acid molecules can self-assemble into micelles by hydrophobic interaction (stage 2). The other factor of importance is the presence of DSPE-PEG in planar bilayers. Even at low ratios of DSPE-PEG (5 mol%) (40), its presence reduces the size of planar bilayers composed of EPC (40) and increases the curvature of the particles. With increasing DSPE-PEG in the bilayer, the PP of mixed micelles decreases and can reach a value of < 0.5 (37). As a consequence, larger vesicles and planar bilayers are transformed into smaller and spherical micelles or cylindrical micelles (36) as shown in Fig. 2a (39,40).

**Fig. 3:**
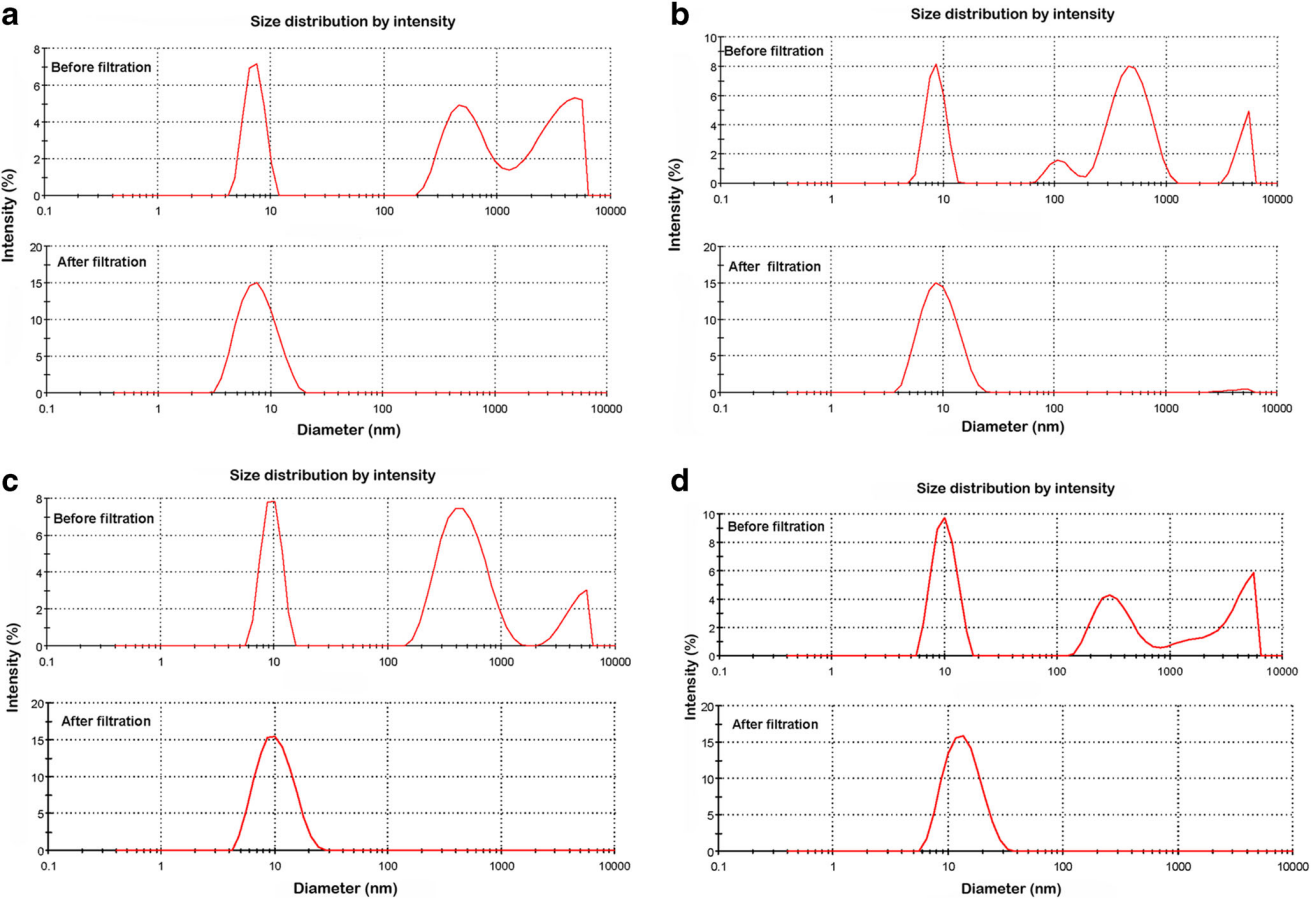
Size intensity weighted diameter of different vitamin K loaded mixed micelles composed of EPC only (**a**), and DSPE-PEG/EPC with molar ratio’s 10/90 (**b**), 30/70 (**c**) and 50/50 (**d**) before and after 3 cycles of filtration, respectively.

After filtration through a membrane with pores of 200 nm, the average sizes of large particles composed of EPC and DSPE-PEG decreased from 213 ± 2 nm, 112 ± 15 nm, 100 ± 21 nm and 132 ± 69 nm to 7.1 ± 0.2 nm, 8.9 ± 0.4 nm, 9.7 ± 0.8 nm and 11.0 ± 1.4 nm (*n* = 3), respectively, for formulations with DSPE-PEG/EPC molar ratios increasing from 0/100 to 50/50 (Fig. 1a). The PDI also reduced substantially from 0.60-0.87 to < 0.26 (Fig. 1b). Figure 3 shows the calculated intensity weighted diameter before and after filtration for formulations with different molar fractions of DSPE-PEG. Before filtration, size distributions by intensity showed that larger particles with diameters above 100 nm were present. Importantly, the intensity of these larger particles (>100 nm) decreased significantly and the intensity of the smaller particles of ~10 nm increased correspondingly after the first filtration. Finally, a fraction of large particles was not detected anymore after 3 filtration cycles and only smaller particles of ~10 nm with a unimodal distribution were present in the dispersions (Fig. 3). Through filtration, larger particles like flat bilayers or vesicles are subjected to both mechanical shear forces when pressed through the pores of the membrane (41) and the high pressure differential (42) across the filter. Spherical mixed micelles are likely being squeezed off from the cylindrical micelles (43) during their passage through the pores (Fig. 2b).

### Lipids Recovery and Vitamin K Loading Capacity

**Fig. 4:**
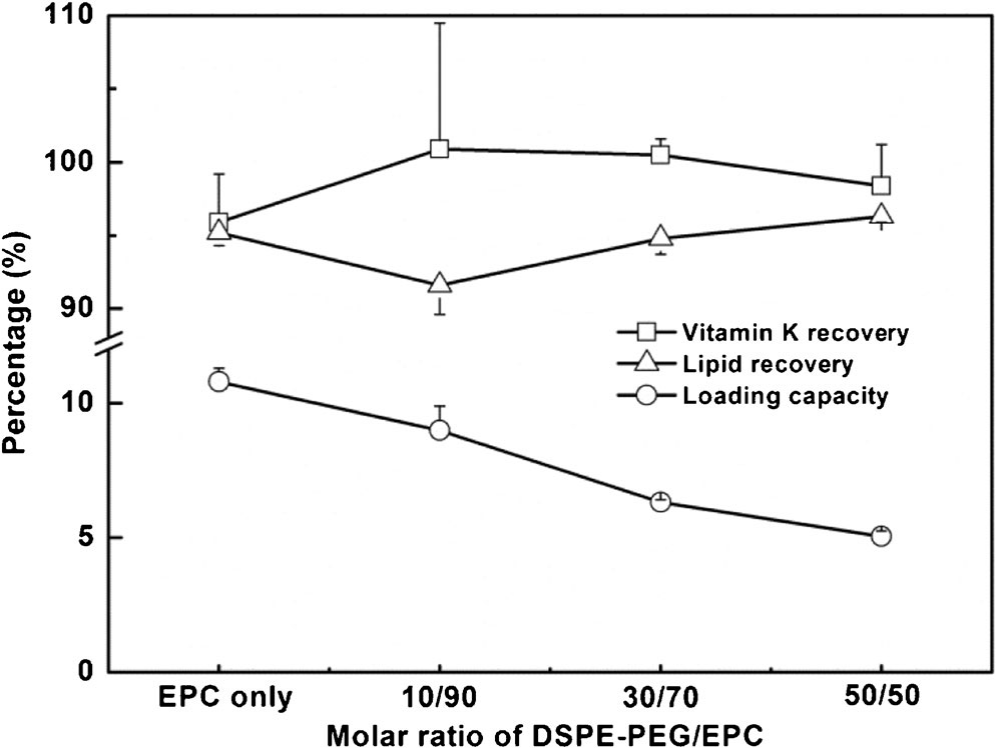
Vitamin K recovery/loading and lipid recovery of mixed micelles composed of different molar ratio of DSPE-PEG/EPC (*n* = 3 independently prepared batches).

The lipid recovery was determined according to Rouser (31). For the mixed micelles with 0.22 molar ratio of vitamin K *versus* total lipids, 91–96% of the lipids was recovered after 3 filtration cycles for the various mixed micelles (Fig. 4).

The vitamin K recovery, defined as the percentage of vitamin K solubilized in the mixed micelles divided by the added amount of vitamin K was 96–100% after three cycles of filtration (Fig. 4). The vitamin K loading capacity of the mixed micelles, defined as the percentage the mass of vitamin K divided by the total mass of the mixed micelles, decreased from 10.8 ± 0.5% to 5.0 ± 0.2% with increasing molar fraction of DSPE-PEG. The reason for this decrease in loading is that the total mass of lipids increased when EPC was replaced by higher-molecular-weight DSPE-PEG 2000.

To get insight into the maximum vitamin K loading capacity of the mixed micelles composed of 50/50 DSPE-PEG/EPC, an increasing amount of vitamin K was added to a fixed amount of total lipids (molar ratio of vitamin K and total lipids was increased from 0.22 to 0.44 and 0.88, respectively). However, in that case, three more filtration cycles were needed to remove large particles with size above 200 nm. Table II shows that both the vitamin K recovery (91.3 ± 2.0%), and the lipid recovery (89.5 ± 4.1%) was high after the 6 successive filtration cycles when the feed of vitamin K was 0.44 (mol/mol lipids). However, vitamin K recovery decreased significantly to 42.6 ± 1.5% and lipid recovery was 41.1 ± 3.9% when the feed of vitamin K was further increased to 0.88 (mol/mol lipids).

**Table II:** Influence of Vitamin K Feed (Molar Ratio of Vitamin K *Versus* Total Lipids) on the Loading Capacity of Mixed Micelles Composed of DSPE-PEG/EPC 50/50 (mol/mol) (*n* = 3 Independently Prepared Batches)

Molar ratio	Vitamin K recovery (%)	Lipid recovery (%)	Loading capacity (w %)	Size (nm)
0.22	98.4 ± 2.8	96.3 ± 0.4	5.0 ± 0.2	11.0 ± 1.4
0.44	91.3 ± 2.0	89.5 ± 4.1	9.9 ± 0.2	19 ± 6
0.88	42.6 ± 1.5	41.1 ± 3.9	18.4 ± 0.5	44 ± 5

**Fig. 5:**
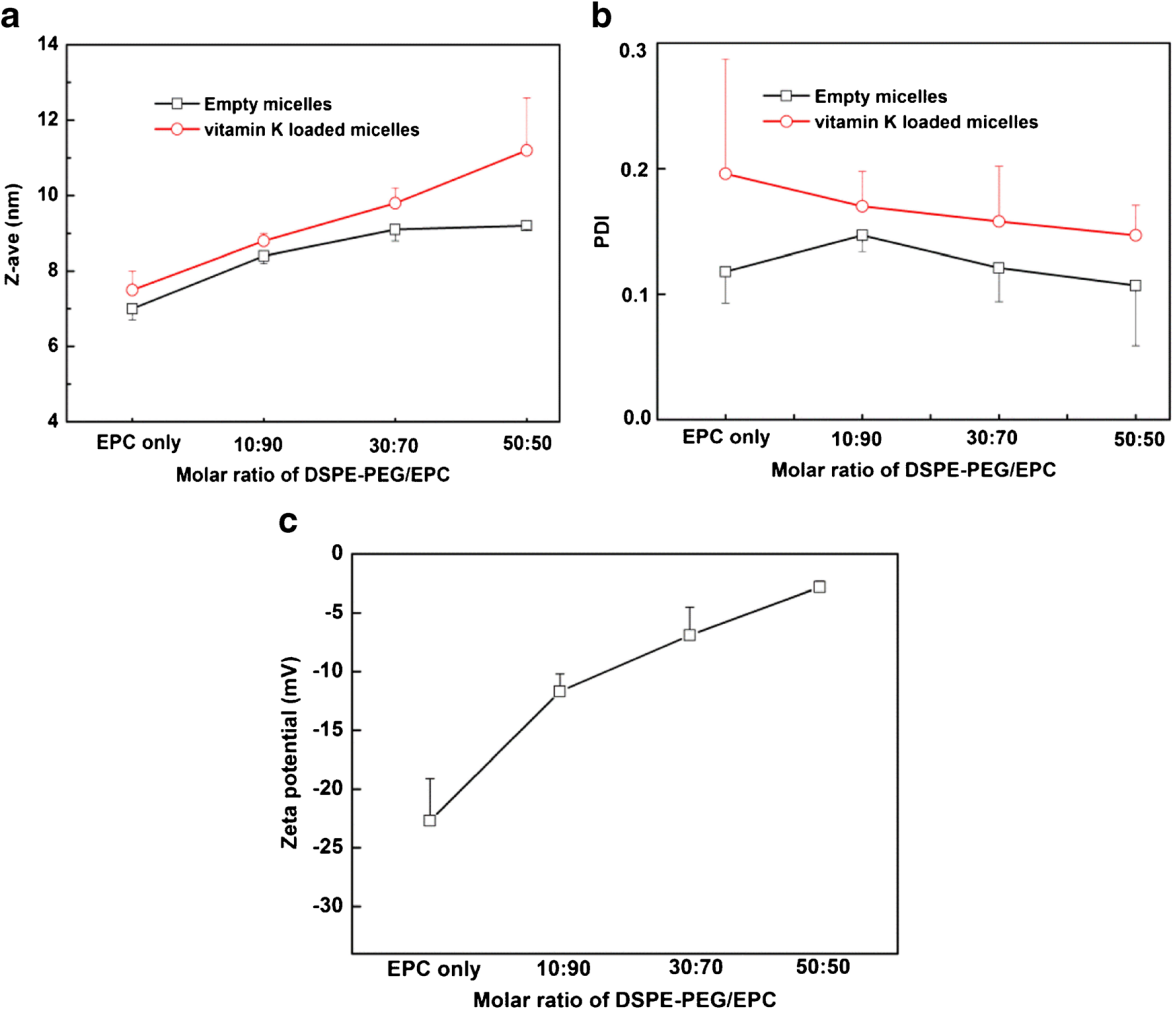
Z-average hydrodynamic diameter (**a**), polydispersity index (PDI) (**b**) and zeta potential (**c**) of mixed micelles with or without vitamin K as a function of molar ratio of DSPE-PEG/EPC (*n* = 3 independently prepared batches).

The size of empty micelles after 3 filtration cycles ranged from 7.0 ± 0.3 to 9.2 ± 0.1 nm with increasing DSPE-PEG content (Fig. 5a). The PDI was < 0.2 for all empty micelles (Fig. 5b). The size of vitamin K loaded micelles (at a feed of 0.22 molar ratio of vitamin K *versus* total lipids) after filtration increased from 7.1 ± 0.2 to 11.0 ± 1.4 nm with increasing DSPE-PEG content (Fig. 5a), which is in good agreement with the average thickness of around 1.5–3.5 nm for PEG corona of DSPE-PEG2000 (22,44). The PDI was <0.2 for all vitamin K loaded micelles (Fig. 5b), which points to a narrow particle size distribution. Figure 5c shows the zeta potentials of the different micelles. The low zeta potential of −22.7 ± 3.6 mV for the mixed micelles composed of bile salt and EPC is caused by deprotonation of the COOH groups of glycocholic acid in phosphate buffer. With an increase of the fraction of DSPE-PEG from 0/100 to 50/50 (mol/mol) in the formulations, an increase of zeta potential from −22.7 ± 3.6 to −2.8 ± 0.5 mV was observed, demonstrating excellent shielding of the surface charge by PEG.

When the feed of vitamin K increased from 0.22 to 0.44, the average size of the mixed micelles increased from 11.0 ± 1.4 to 19 ± 6 nm after 6 times filtration, because still a small fraction of bigger particles between 100 and 200 nm was present even after 6 filtration cycles. The majority of particles however had an average size of around 15 nm (Supplementary Fig. 1a). When the feed of vitamin K further increased from 0.44 to 0.88 (mol/mol lipids), the micellar dispersions needed to be diluted to avoid occlusion during the filtration. The average size of the mixed micelles ended up at 44 ± 5 nm after the six filtration cycles. The average size of a minor fraction of micelles was around 18 nm but the majority had a size of between 100 and 200 nm (Supplementary Fig. 1b).

**Fig. 6:**
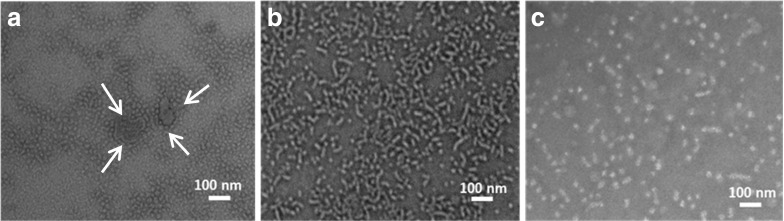
TEM images of vitamin K loaded mixed micelles composed of DSPE-PEG/EPC 50/50 and a vitamin K feed molar ratio of 0.22 (mol/mol lipids): before filtration (**a**), after the first filtration cycle (**b**) and after 3 filtration cycles (**c**).

The morphology of the mixed micelles composed of DSPE-PEG/EPC 50/50 (mol/mol) with vitamin K feed molar ratio of 0.22 (mol/mol lipids) was investigated by TEM analysis (Fig. 6). Figure 6a (arrows) shows several large particles of approximately 150 nm before filtration which confirmed the size as determined by DLS (Fig. 3d). After the first filtration cycle, cylindrical structures of up to approximately 100 nm in length can be seen besides spherical micelles (Fig. 6b), which are common shapes of mixed micelles composed of phospholipids and bile salts (40). One explanation for the formation of cylindrical structures is that TEM images show the shape of mixed micelles in their dry state. Thus, during the evaporation of water for the preparation of the specimen, increasingly higher concentrations of electrolytes such as phosphate buffer screen the electrostatic repulsion and promote particle aggregation (45). Also, PEG can induce lipid fusion in the dry state (46,47), finally resulting in cylindrical structures as shown in Fig. 6b. Importantly, after 3 filtration cycles, although the effect of drying on the shape of micelles can also be observed in Fig. 6c, in which several small mixed micelles fused into pearl-necklace-like structures are seen (48), mostly spherically shaped mixed micelles with diameter around 10 nm were present in the sample of Fig. 6c.

Like for conventional surfactants, the formation of mixed micelles can be demonstrated using the fluorescent probe pyrene. The pyrene fluorescence intensity ratio of I_338_/I_333_ is plotted as a function of concentration of glycocholic acid in Supplementary Fig. 2. The CMC of mixed micelles composed of EPC only was 4.6 μM glycocholic acid. For mixed micelles composed of DSPE-PEG/EPC (50/50), their CMC was 3.9 μM glycocholic acid. Within the experimental error, the CMCs of both mixed micelles are equal.

### Stability and Vitamin K Retention at Acid pH

To study the stability of various mixed micelles, size changes were measured when the pH was stepwise decreased from 7.3 to 1.6 and subsequently reversed to 7.3. The size of micelles composed of EPC only was 80 nm after the pH was lowered to 4.2 (Supplementary Fig. 3a), while turbidity appeared after the pH was further lowered to 3.5 (Supplementary Fig. 4a). Apparently, the micelles became unstable and aggregated or coalesced, resulting in precipitation of vitamin K (Supplementary Fig. 4b). The pH of the dispersions was then stepwise lowered to 1.6 and they were subsequently incubated for 1 h at 37°C. After centrifugation, the amount of vitamin K in the supernatants as measured by HPLC was found to be only 1.5 ± 0.1 and 6.3 ± 0.3% of the initial vitamin K loading for the commercial formulation and mixed micelles composed of EPC only, respectively (Supplementary Fig. 5).

**Fig. 7:**
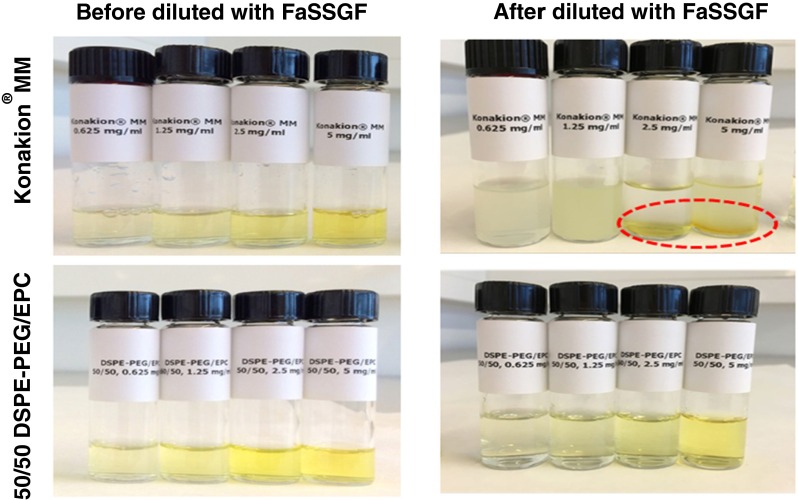
Photographs of mixed micelles composed of EPC only (Konakion® MM) and 50/50 DSPE-PEG/EPC (mol/mol) with different vitamin K concentrations (from 0.625 to 5 mg/ml), incubated for 1 h at 37°C with and without FaSSGF.

For mixed micelles composed of DSPE-PEG/EPC 10/90 (mol/mol), their size increased from 9 to 100 nm and dispersions became translucent, when the pH was lowered stepwise to 1.6 (Supplementary Fig. 3b). It was further measured that 58.2 ± 0.9% of vitamin K remained solubilized in the mixed micelles (Supplementary Fig. 5). Mixed micelles composed of DSPE-PEG/EPC 30/70 (mol/mol) remained transparent and the size increased only slightly from 10 to 20 nm when the pH was lowered from 7.3 to 1.6 (Supplementary Fig. 3c). The size remained around 20 nm (with PDI at around 0.2) when the dispersions were kept at pH 1.6 at 37°C for 80 mins (Supplementary Fig. 3e), and around 90% of vitamin K remained solubilized at pH 1.6 (Supplementary Fig. 5). Interestingly, the size of the mixed micelles composed of DSPE-PEG/EPC 50/50 (mol/mol) did not show any change when the pH was lowered from 7.3 to 1.6 and *vice versa* (Supplementary Fig. 3d) and remained around 12 nm with PDI of ~0.2 at pH 1.6 and 37°C for 80 min (Supplementary Fig. 3f), while more than 90% of vitamin K remained solubilized at pH 1.6 (Supplementary Fig. 5). The stability of mixed micelles was further investigated in FaSSGF (pH 1.5). Konakion® MM was not stable and coalescence of vitamin K was observed within 1 h at 37°C (Fig. 7). However, mixed micelles composed of 50/50 DSPE-PEG/EPC (mol/mol) remained stable and kept transparent in FaSSGF for at least 1 h at 37°C. To conclude, due to the steric stabilization of mixed micelles by PEG, the formation of aggregated particles can thus be avoided by introducing PEG in the formulation.

### Cell Viability Study

**Fig. 8:**
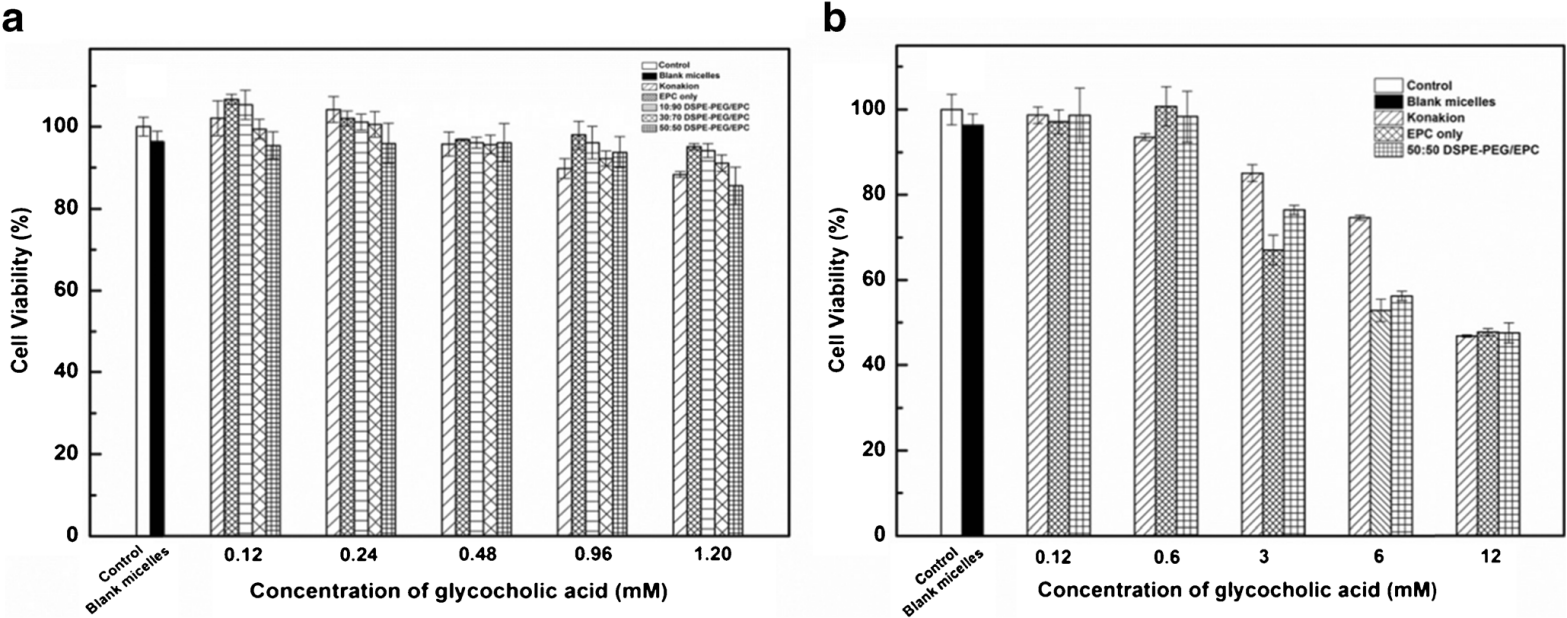
*In vitro* cell viability of Caco-2 cells incubated for 24 h with mixed micelles composed of EPC only and different molar ratio of DSPE-PEG/EPC (from10/90 to 50/50, mol/mol) and Konakion® MM.

The effect of vitamin K loaded mixed micelles of different compositions as well as the commercial formulation Konakion® MM on the viability of Caco-2 cells was studied using the XTT assay. Caco-2 cells were selected as a model for small intestinal epithelial cells (49,50). As shown in Fig. 8a, the cell viability after 24 h of incubation with different formulations with concentration of glycocholic acid ranging from 0.12 to 1.20 mM was > 90%. Figure 8b also shows the *in vitro* cell viability of Caco-2 cells incubated with commercial formulation Konakion® MM and mixed micelles composed of EPC only or DSPE-PEG/EPC (50/50 mol/mol) at higher concentrations of glycocholic acid (from 0.12 to 12 mM) for 24 h. Cell viability decreased from 95 to 50% when the concentration of glycocholic acid increased from 0.12 to 12 mM, respectively. The reduced cell viability is due to high concentration of glycocholic acid (33,51). Therefore, mixed micelles composed of DSPE-PEG/EPC showed good cytocompatibility which is comparable with that of the commercial formulation Konakion® MM.

## CONCLUSIONS

In this study, vitamin K loaded mixed micelles composed of DSPE-PEG, EPC and glycocholic acid were successfully prepared by a film hydration method. Extrusion through a membrane filter plays a crucial role in preparation of mixed micelles with final sizes at around 10 nm. Vitamin K loaded mixed micelles composed of EPC, DSPE-PEG 2000 and glycocholic acid showed high loading capacity and good cytocompatibility for Caco-2 cells, which was comparable to mixed micelles without DSPE-PEG (*i.e.* Konakion® MM). Additionally, mixed micelles of EPC, DSPE-PEG 2000 and glycocholic acid can prevent both the formation of larger aggregates and coalescence of vitamin K in the micellar dispersions at low pH thus is superior to Konakion® MM with regard to their gastric stability. Therefore, mixed micelles of EPC, DSPE-PEG 2000 and glycocholic acid are a promising oral formulation of vitamin K for the prophylaxis and treatment of VKDB in neonates and infants suffering from cholestasis. More work about the intestinal transport of vitamin K loaded mixed micelles composed of EPC, DSPE-PEG 2000 and glycocholic acid will be conducted in the near future.

## Electronic supplementary material

Below is the link to the electronic supplementary material.

ESM 1 (DOCX 1385 kb)

## ABBREVIATIONS

EPC: Egg phosphatidylcholine
DSPE-PEG 2000: 1,2-distearoyl-sn-glycero-3-phosphoethanolamine-N-[methoxy(polyethyleneglycol)-2000]
TEM: Transmission electron microscopy
DLS: Dynamic light scattering
CMC: The critical micelle concentration
VKDB: Vitamin K deficiency bleeding
MM: Mixed micelles
PP: Packing parameter
V: Molecular volume
A_o_: Polar surface cross-area
L: Length of the hydrophobic chain
FaSSGF: Fasted state simulated gastric fluid
PMS: *N*-Methyl dibenzopyrazine methylsulfate
XTT: Sodium 3′-[1-(phenylaminocarbonyl)-3,4-tetrazolium] -bis(4-methoxy-6-nitro)benzene sulfonic acid hydrate)
