# Supplementary material for: A Mixed Micelle Formulation for Oral Delivery of Vitamin K
Source: Pharm Res. 2016 May 31;33:2168–79. doi: 10.1007/s11095-016-1954-9 (PMC4967097; doi:10.1007/s11095-016-1954-9)
Supplement: Supplementary file 1 — (DOCX 1385 kb) [file 11095_2016_1954_MOESM1_ESM.docx]

**A Mixed Micelle Formulation for Oral Delivery of Vitamin K**

Feilong Sun^1^, Tessa Jaspers^1^, Peter M. van Hasselt^2^, Wim E. Hennink^1^, Cornelus F. van Nostrum^1,^*

^1^ Department of Pharmaceutics, Utrecht Institute for Pharmaceutical Sciences, Utrecht University, 3584 CG Utrecht, The Netherlands

^2^ Department of Pediatrics, Wilhelmina Children's Hospital, University Medical Center Utrecht, Lundlaan 6, 3584 EA Utrecht, The Netherlands

Corresponding Author

*Tel.: + 31 620274607. Fax: + 31 30 251 7839. E-mail: C.F.vanNostrum@uu.nl

**Supplementary material**


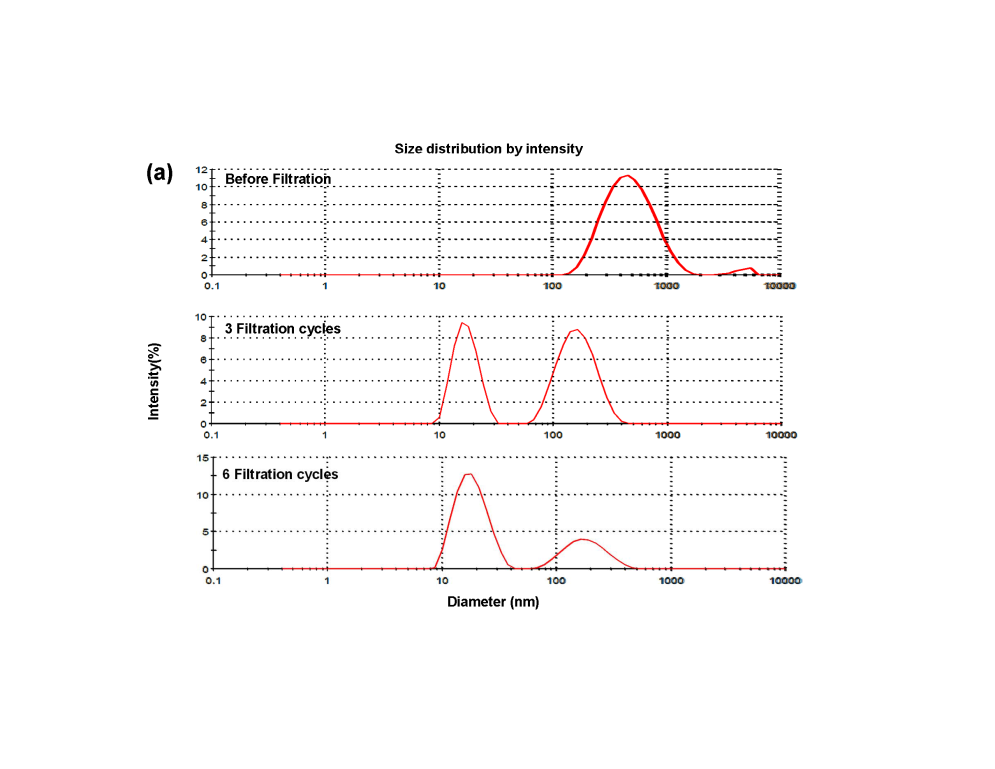

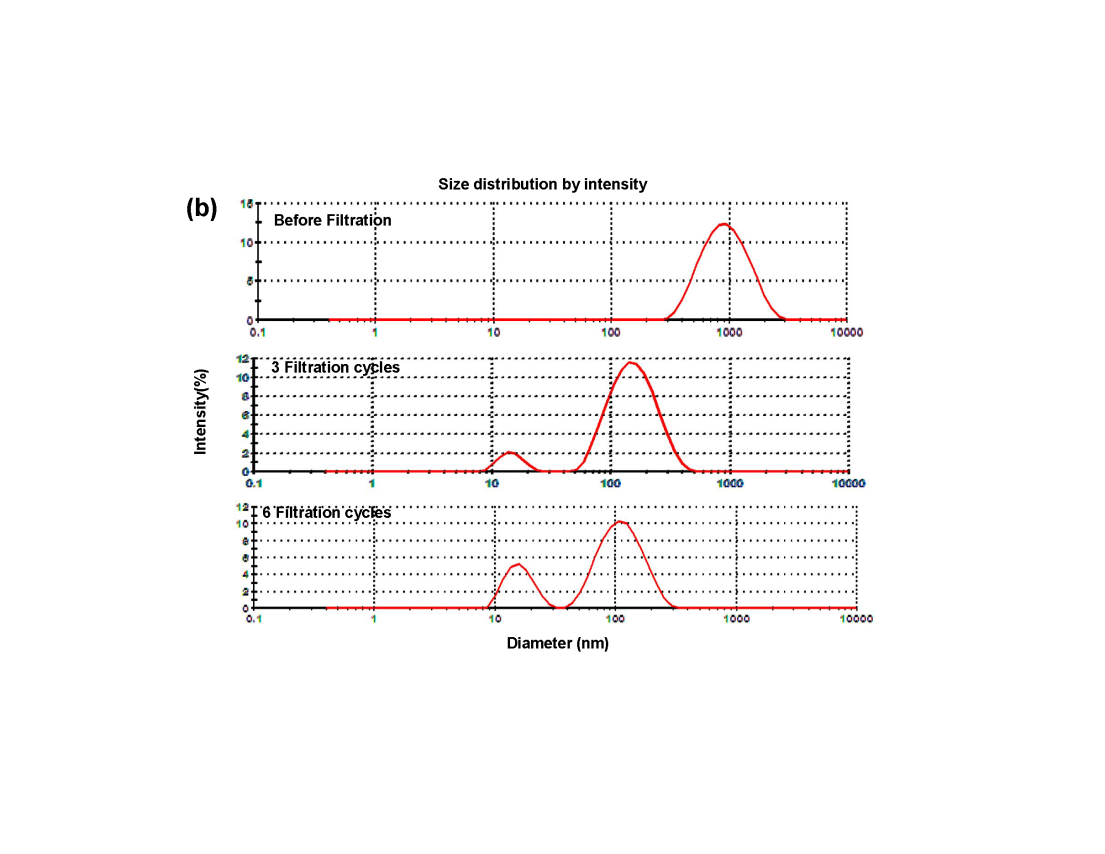


Fig. 1 Size intensity weighted diameter of vitamin K loaded mixed micelles composed of DSPE-PEG/EPC 50:50, (mol/mol) before filtration, after 3 filtration cycles and after 6 filtration cycles with the feed of vitamin K 0.44 (b) and 0.88 mol/mol lipids (c), respectively.

.





Fig. 2 The fluorescence intensity ratio I_338_/I_333_ of pyrene as a function of the concentration of glycocholic acid in the micelles composed of EPC only (a) and DSPE-PEG/EPC 50:50 (b)


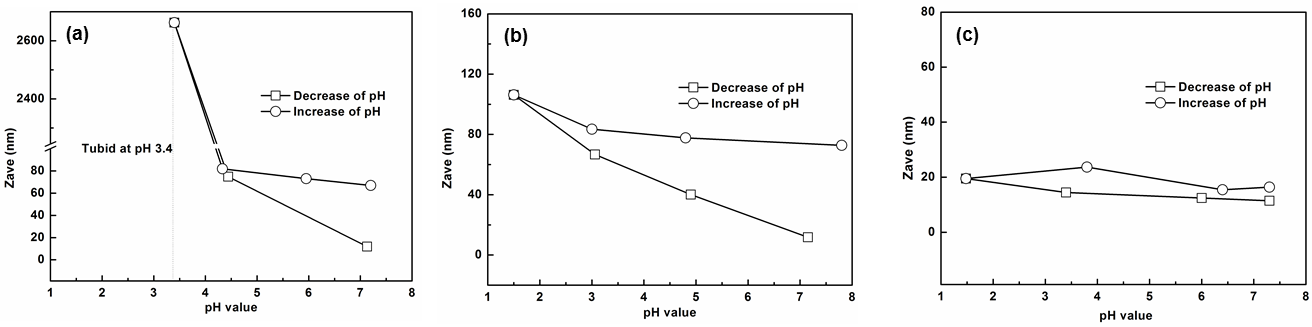


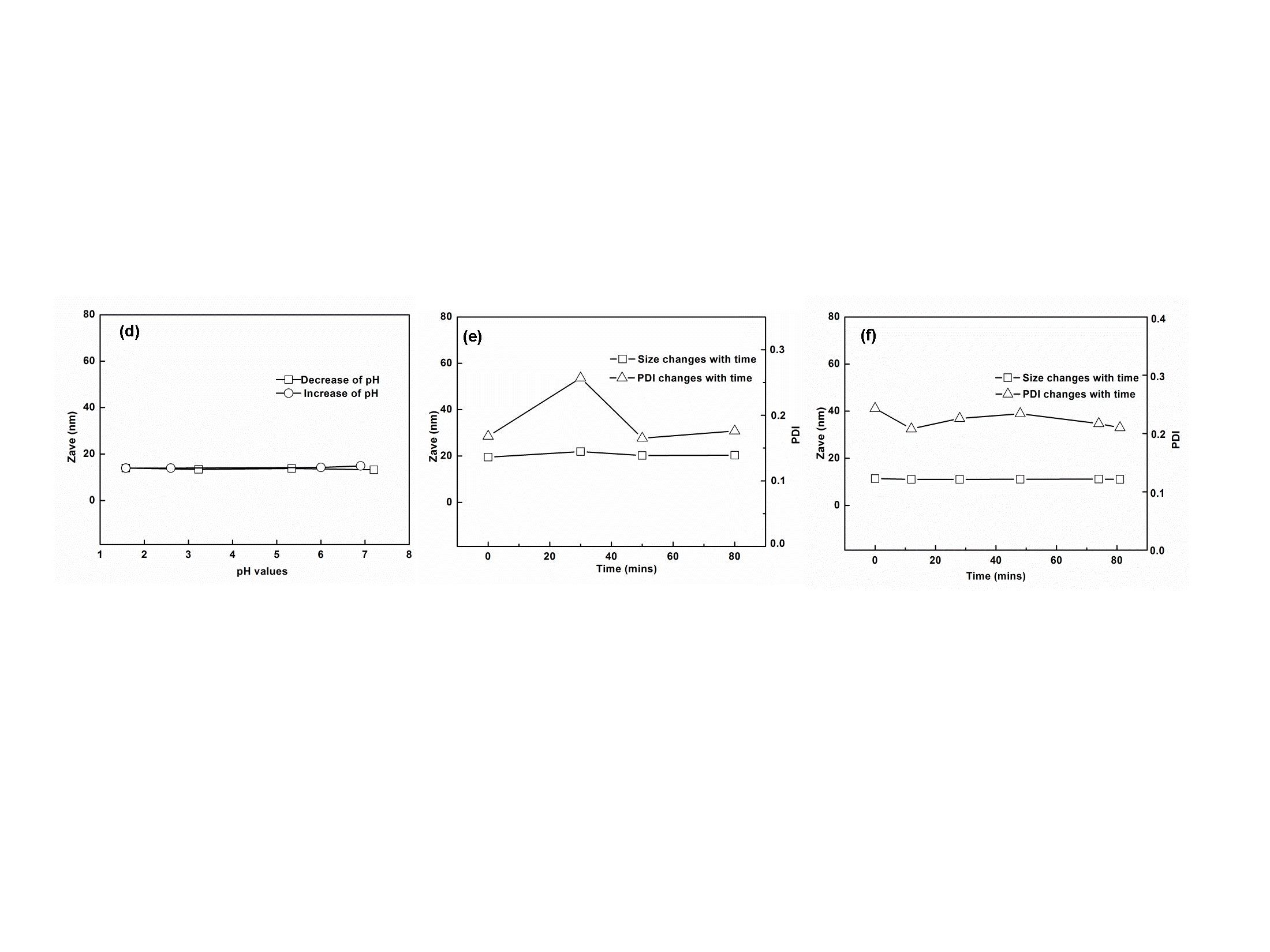


Fig. 3 pH stability study by DLS: Z-average diameter at room temperature with decreasing and subsequent increasing pH for vitamin K loaded mixed micelles composed of EPC only (a), and various molar ratio of DSPE-PEG/EPC (mol/mol) 10/90 (b), 30/70 (c) and 50/50 (d), respectively. Size changes with time at pH 1.6 and 37°C for vitamin K loaded mixed micelles composed DSPE-PEG/EPC (mol/mol) 30/70 (e) and 50/50 (f).


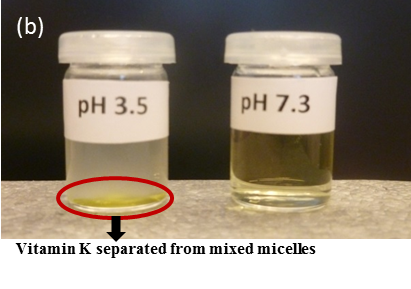


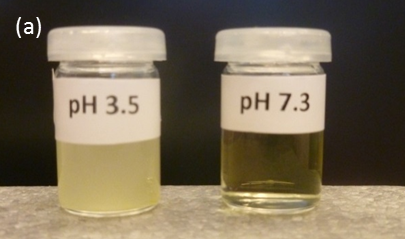


Fig. 4 Photographs of Konakion MM composed of EPC only incubated at 37°C for 0 hour (a) and 1 hour (b) at pH 3.5 and pH 7.3, respectively.





Fig. 5 Vitamin K recovery in the supernatant of micellar dispersions after incubation for 1 hour at pH 1.6 (n=3 independently prepared batches)
